# Influence of Different Solvents and High-Electric-Field Cycling on Morphology and Ferroelectric Behavior of Poly(Vinylidene Fluoride-Hexafluoropropylene) Films

**DOI:** 10.3390/ma14143884

**Published:** 2021-07-12

**Authors:** Till Mälzer, Lena Mathies, Tino Band, Robert Gorgas, Hartmut S. Leipner

**Affiliations:** 1 Interdisciplinary Center of Materials Science, Martin Luther University Halle-Wittenberg, Heinrich-Damerow-Straße 3, 06099 Halle, Germany; lena.kuske@cmat.uni-halle.de (L.M.); hartmut.leipner@cmat.uni-halle.de (H.S.L.); 2 enspring GmbH, Weinbergweg 23, 06120 Halle, Germany; robert169@web.de; 3 Institute of Physics, Martin Luther University Halle-Wittenberg, Von-Danckelmann-Platz 3, 06099 Halle, Germany; tino.band@physik.uni-halle.de

**Keywords:** ferroelectric polymers, P(VdF-HFP), solvents, high-electric-field cycling

## Abstract

P(VdF-HFP) films are fabricated via a solution casting doctor blade method using high (HVS) and low (LVS) volatile solvents, respectively. The structural properties and the ferroelectric behavior are investigated. The surface structure and crystal phase composition are found to be strongly dependent on the type of solvent. LVS leads to a rougher copolymer surface structure with large spherulites and a lower crystallinity in contrast with HVS. The crystalline phase of copolymer films fabricated with HVS consists almost exclusively of α-phase domains, whereas films from LVS solution show a large proportion of γ-phase domains, as concluded from Raman and X-ray diffraction spectra. Virgin films show no ferroelectric (FE) switching polarization at electric field amplitudes below 180 MV/m, independent of the solvent type, observed in bipolar dielectric displacement—electric field measurements. After applying electric fields of above 180 MV/m, a FE behavior emerges, which is significantly stronger for LVS films. In a repeated measurement, FE polarization switching already occurs at lower fields. A shielding effect may be related to this observation. Additionally, Raman bands of polar γ-phase increase by high-electric-field cycling for the LVS sample. The solvent used and the resulting crystal phase composition of the virgin sample is crucial for the copolymer behavior during bipolar electrical cycling.

## 1. Introduction

Polymer dielectric films are of particular importance for energy storage applications, such as film capacitors and electrolyte layers in secondary batteries. The hydrophobic copolymer poly(vinylidene fluoride-co-hexafluoropropylene) (P(VdF-HFP)), with its intrinsic properties including high solubility in many solvents, good mechanical, chemical, and thermal stability, and low electron conductivity [1], is widely used for thin polymer layers in sensors, energy storage devices and medical applications [2]. As a polymer matrix, it can be modified with different additives according to the desired application [3].

P(VdF-HFP)-based films show a semi-crystalline structure. The degree of crystallinity of the polymer, which means the fraction of ordered molecules, can be reduced by increasing the fraction of HFP, improving the solubility in different solvents [4], and facilitating the processing. The crystalline phase is formed by the P(VdF) monomers [5,6] that crystalize in different phases [7]. The P(VdF) monomer shows a relatively high dipole moment due to the alternating CF_2_ and CH_2_ groups and the large difference in electronegativity between H and F.

The most important crystalline phases are the nonpolar α-phase, with a TGTG- (trans, gauche, trans, minus gauche) chain conformation (form II) with an antiparallel packing of the chains that leads to a nonpolar unit cell, and the polar β- and γ-phase (form I and III), consisting of all trans and TTTGTTTG- chain conformations, respectively [7,8,9,10]. There are further crystalline phases with the same chain conformations as the ones stated above but different chain stackings [9,11]. The polar β-and γ-phases are both electrically active. The β-phase shows the highest dipolar moment per unit cell [2] and strong ferroelectric (FE) behavior [12]. Depending on the process parameters and additional heating, different phases can be targeted in the manufacturing process [2,13,14,15]. Although the polar phases are thermodynamically preferred, the α-phase is kinetically favored [16]. The final phase composition formed during the drying process can be controlled by knowing the solubility curves (concentration of polymer in the solvent versus temperature) of the distinct phases, which depends strongly on the kinetics of formation, growth, and transformation of the crystal phases [17]. Many studies focus on the different crystalline phases of P(VdF), their identification and properties [7,8,14,18], as well as the targeted production of individual phases via post-treatment such as annealing, electrical treatment, or stretching [2,13,19,20,21].

On a laboratory scale, films are often produced from solutions. The easy processing and highly scalable methods offer great advantages in the industrial use of the material. Despite its toxic properties, dimethylformamide (DMF) is one of the commonly used solvents for P(VdF)-based copolymer film preparation [13,14,22]. Furthermore, the structurally similar dimethylacetamine (DMA) with equivalent harmful properties is widely used [7,8,15,16,23]. Using high boiling point solvents that are fully miscible with water such as the two previously mentioned P(VdF) films tend to have a porous and rough surface. Films must be fabricated at low relative humidity or at high substrate temperature to receive smooth films [24]. Alternatively, polar solvents with a lower boiling point can be used, e.g., methyl ethyl ketone (MEK) [18], acetonitrile [25], and acetone [5,8,26] with good dissolving properties (whereby partly increased temperatures are needed) and that are harmless to health.

In this work, we present a detailed study on the morphological, structural, and electrical properties of P(VdF-HFP) films processed from solutions of different solvents in a doctor blade casting machine under controlled and reproducible drying conditions. We compare the effect of acetone, MEK, DMF, and N-methyl-2-pyrrolidone (NMP) as solvents on the properties of the manufactured P(VdF-HFP) films. The use of harmless solvents in combination with the reproducible and easily upscalable doctor blade casting process offers opportunities for the industrial fabrication of this material class. Using solvents like acetone or MEK with a lower boiling point and more volatility than DMF and NMP, we could process films with a fine surface structure. The solvents used have a strong impact on the crystallinity and the resulting crystalline structures in the films. In this work, we investigate the influence of the solvents on the morphology and structure of the virgin samples and the influence of bipolar electrical cycling on the films’ FE behavior and phase composition.

## 2. Materials and Methods

### 2.1. Material Preparation

The P(VdF-HFP) copolymer with an HFP content of minimum 5 wt.% and a molecular mass of Mw ~400 kg/mol, Mn ~130 kg/mol from Sigma–Aldrich, and the solvents NMP from VWR Chemicals, DMF from Sigma–Aldrich, MEK from VWR Chemicals and acetone from Micro Chemicals were used as received. P(VdF-HFP) was mixed with the solvent and stirred at room temperature (at 50 °C for MEK solutions) for at least for 6 h to dissolve the copolymer. The copolymer’s different solvation properties, especially for MEK, have been attributed to the different solubility parameters of solvents corresponding to the Hansen solubility parameter model [27] (see Supplementary Materials for detailed information). The P(VdF-HFP) solutions were cast on aluminum foil substrates using a doctor blade method. The film drying process was conducted immediately after deposition onto a heatable process table, an infra-red lamp, and a convection dryer. The drying conditions were adjusted to the distinct properties of the different solvents (Table 1) to achieve homogeneous and nonporous films. The drying dwell times were extended after the samples appeared optically dry to ensure total evaporation of the solvents (Table 2). We use the categorization of HVS for acetone and MEK and LVS for DMF and NMP in the following graphs.

### 2.2. Structural Characterization

Scanning electron microscopy (SEM) was performed with a XL30 ESEM FEG (FEI/Philips, Waltham, MA, USA) taking secondary electron (SE) images of the surfaces. The surface roughness was analyzed by atomic force microscopy (AFM) in height and phase contrasts with a Nanoscope Dimension 3000 (Bruker Corp./Digital Instruments/Veeco Metrology, Billerica, MA, USA) and a silicon NCL-tip (Nanosensors) with a tip radius of <10 nm. Different scan areas with a size of 20 × 20 µm^2^ were investigated, and a representative measurement was evaluated in detail. Thermal analysis (differential scanning calorimetry, DSC) was done with a Differential Scanning Calorimeter Model DSC7 (Perkin Elmer, Waltham, MA, USA). 13–15 milligrams of the copolymer films were cut into small pieces, filling aluminum pans; two cooling/heating runs were then performed with a ramp of 10 K/min in a nitrogen atmosphere between −80 °C and +200 °C. X-ray diffractometry (XRD) was performed in transmission mode with a multipurpose diffractometer system type STADI MP (STOE & Cie. GmbH, Darmstadt, Germany), with a MYTHEN 1K detector (DECTRIS AG, Baden-Daettwil, Switzerland) and Mo_Kα_ radiation (λ = 0.7093 Å). Raman spectra were measured with a LabRam Raman microscope (Horiba Europe GmbH/Dilor, Oberursel, Germany) with an 1800 groove/mm grating and a spectral resolution of 1 cm^−1^, equipped with a 632.8 nm HeNe-laser (30 mW) and a 100-fold magnification lens (spot size of 1 µm in diameter). Raman spectra of 100 points with an equal distance within a square of 20 × 20 µm^2^ were recorded for each sample; the mean spectrum of the normalized spectra is used for presentation and discussion. The ratio of specific band intensities was calculated separately for each point, and the mean value and standard deviation from averaging are presented and used for discussion. In order to investigate the influence of bipolar electrical cycling on the polymer phase composition, the samples were first electrically treated using the Al substrate as the bottom and sputtered Al disks with 2.3 mm in diameter as the top electrodes. After electrical cycling, the Al substrate was removed, and Raman spectra were collected from untreated (virgin) and bipolar electrical treated areas of the samples.

### 2.3. Electrical Characterization

For electrical characterization, a copolymer film area of 3 × 3 cm^2^ was cut out and peeled off the substrate. Aluminum electrodes with a thickness of 500 nm and a diameter of 2.3 mm were magnetron sputtered onto both film surfaces. For the analysis of FE behavior, bipolar electric displacement field-electric field (*D-E*) characteristics were investigated under ambient conditions. The measurement of *D-E* loops was conducted using a Sawyer-Tower circuit modified to the virtual ground mode [28], which is based on a charge measurement. The charge measurement is essentially conducted via an integrating amplifier as a current to charge converter. The integrating amplifier consists of an operational amplifier and a linear known-valued feedback capacitor connected between the amplifier’s output and the noninverted terminal. With this setup, the dielectric breakdown was measured with an applied voltage ramp of 80 V/s. Cyclic bipolar *D-E* loops were examined with a triangular field sweep at 1 Hz with various amplitude maxima of the electric field up to 283 MV/m. For each electric field amplitude, a maximum of 10 measurement cycles were conducted. Electric field amplitude maximum was gradually increased in steps of 33 MV/m and starting at 50 MV/m. After cyclization with the highest field amplitude maximum, a complete run (R) of bipolar electrical (BE) cycling was completed. To investigate changes in *D-E* characteristics due to BE cycling or sample storing after a cycling run, repetitions of BE cycling runs were conducted. The samples were covered with silicone oil to prevent corona discharge during the high-electric-field measurements.

## 3. Results

### 3.1. Structural Properties

**Figure 1:**
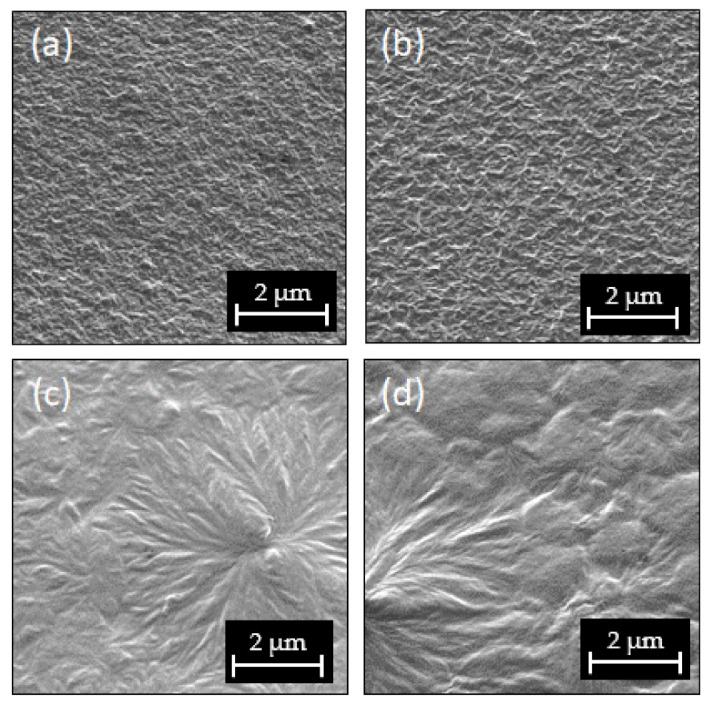
Secondary electron images of films processed from different solvent solutions: (**a**) Acetone; (**b**) MEK; (**c**) DMF; (**d**) NMP.

The preparation under controlled drying conditions leads to free-standing films with a thickness of approximately 6 μm (±0.5 μm). The films show distinct surface structures in scanning electron microscopy (SEM) images (Figure 1). Due to the more volatile solvents, acetone, and MEK, and despite the drying conditions adjusted, the drying of the processed films from these solutions is much faster than for DMF and NMP solutions. Films processed from high volatile solvents (HVS) (acetone, MEK) led to a fine surface structure (Figure 1a,b) with no long-range order. Films from the DMF solution show spherulites with a diameter of a few micrometers and a radial structure of lamellar crystallites (Figure 1c). The high boiling point of NMP and the slower drying of the films result in a hilly but smooth surface structure, interrupted by lamellae and some spherulites, similar to those in the samples from DMF solution (compare with Figure 1c,d). Atomic force microscopy (AFM) images confirm the SEM results. Surface roughness determined by AFM is shown in Table 3 and Figures S1 and S2. In the film processed from NMP, the solvent with the highest boiling point, the islands forming the hilly surface show a low surface roughness. The slow drying of the samples from low volatile solvents (LVS) led to a long-range order resulting in the formation of larger (semi)crystalline structures, whereas the number of small crystallites present in the HVS samples appeared to be reduced. The overall surface roughness is much higher in the films processed from LVS, matching the much larger crystal structures.

**Figure 2:**
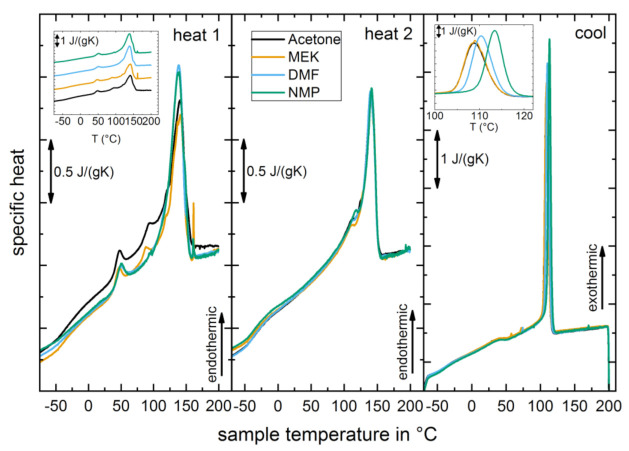
DSC thermograms of P(VdF-HFP) films processed from different solvents. Heating run 1 (**left**), heating run 2 (**center**), and cooling run (**right**).

For thermal analysis, differential scanning calorimetry (DSC) measurements were evaluated. The thermograms of all samples differ only slightly for the first heating run (Figure 2). The film processed from the NMP solution shows an increased specific heat that only aligns towards the other three curves after reaching 200 °C once, which indicates that NMP was not fully evaporated during the drying process of the copolymer films, and residual solvent remained in the copolymer film, related to the high boiling point of 204 °C. There was no indication of residual solvent in the other samples. The position of the main melting peak is similar for all samples (around 140 °C). The thermograms do not provide a clear indication of different crystalline phases. Compared to pure P(VdF), the melting peak is about 30 °C lower [2], which can be attributed to the effect of HFP comonomer [29,30,31,32]. The slight shift in the melting peak of 2–3 °C towards higher temperatures for the samples processed from LVS (DMF, NMP) may be due to an increase in lamellar thickness and degree of molecular order [33,34,35]. Different crystal phases can also lead to different peak positions with the commonly discussed higher melting temperature for the PVDF γ-phase [2,33,34,35]. Furthermore, endothermic peaks in the thermogram of films from LVS at approximately 88 °C, 120 °C, 93 °C, and 121 °C for samples from DMF and NMP solution, respectively, are attributed to temperatures induced by the films due to the drying process parameters [33]. These peaks vanish in the thermogram of heating run 2 (Figure 2). An additional small endothermic peak appears at around 50 °C in equal shape for all samples whose origin is unclear. The glass transition temperature (*T*_g_) was at –37 °C for all samples, being in good agreement with other experimental studies of P(VdF-HFP) [36,37]. For heating run 1, the melting enthalpies *H*_meas_ determined are approximately 40 J/g and 33 J/g for the HVS samples and DMF, respectively (a straight line from 68 °C to 160 °C was used as a baseline for integration). The crystallinity can be estimated by taking the measured enthalpies in relation to the melting enthalpy of pure crystalline P(VdF) *H*_c_, with mass correction, as there are 6 wt.-% HFP monomers in the film samples. *H*_c_ is 98 J/g as the mean value of mentioned enthalpies in Lovinger et al. (93 J/g and 103 J/g for α-phase and β-phase of PVDF, respectively) [35]. The degree of crystallinity, *c* = *H*_meas_/(0.94 * *H*_c_), is 0.43 and 0.36 for HVS films and DMF, respectively; this indicates a 7% points higher degree of crystallinity for the HVS samples and can be attributed to the mass crystallization effect taking place due to the high evaporation rate and short drying time.

**Figure 3:**
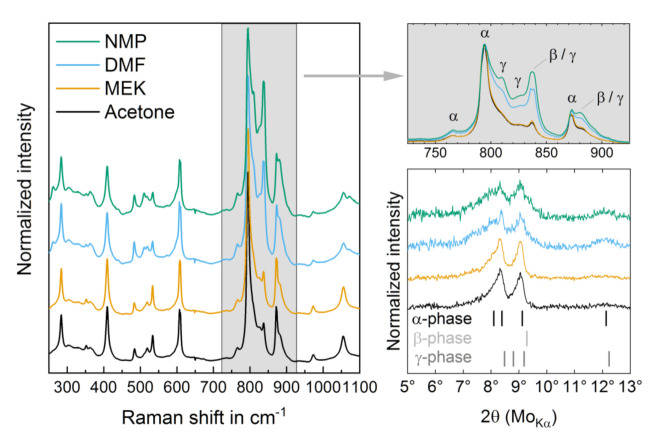
Raman spectra of the virgin P(VdF-HFP) films processed from different solvents (**left**). Detailed view of the representative region of the Raman spectra (b) with band assignments for main phases of P(VdF) [2,8,10]. All Raman spectra are normalized to the highest band at 795 cm^−1^, characteristic of the α-phase. XRD of the different copolymer films processed from different solvents (**bottom right**). Marked positions for reflexes are expected for the three main phases of P(VdF) [2]. The graphs are normalized to the main reflex at 8.3° for better comparison.

The copolymer films show low intensity signals in X-ray diffractometry (XRD) measurements (Figure 3, bottom right) due to the light elements. As a result of the semicrystalline structure, the reflexes are broad. It can be deduced from the position of the reflexes that the crystalline areas of the films predominantly contain the P(VdF) α-phase [2,38]. Samples processed from LVS seem to have a worse signal-to-noise ratio, which may be assigned to a lower degree of crystallinity. In the XRD measurements, there is no indication for a significant amount of β-phase in the samples. Still, the diffraction pattern of the LVS samples shows a changed reflex pattern indicating a changed composition of crystalline P(VdF) phases. Reflexes at approximately 8–10° are separated from each other less clearly and show a slightly different intensity ratio, whereas the reflex at 12° gains intensity.

The Raman measurements (Figure 3) support the idea that HVS samples promote the formation of the kinetically favored α-phase of P(VdF). The Raman spectra of these samples are in good agreement with published spectra of α-phase samples [39,40]. For the HVS samples, the averaged measurements are congruent, indicating a homogeneous surface structure. For the LVS samples, the measurements show a slight dispersion, which can be explained by a variety of crystalline structures in distinct areas of the film, like lamellar parts, spherulites, and smooth areas. In Figure 3 left, the Raman spectra are plotted. The HVS samples show a strong characteristic signal at 795 cm^−1^, resulting from CH_2_ rocking modes in the nonpolar α-phase of P(VdF) [8]. In the HVS samples, the process of drying the films was completed fast (within 1 min) and resulted in quenching the copolymer solution and many small crystallites of the kinetically favored α-phase of P(VdF), in accordance with other studies [14,33]. In the LVS samples, the intensity ratios of the signals change (see Figure 3, left and top right, and Table 4). The higher intensities of the bands at 261, 510, 837, 881 and 1072 cm^−1^, and the shoulders at 810 and 826 cm^−1^, indicate a different copolymer structure in these films. The majority of these bands can be attributed to the polar β- or γ-phases of P(VdF) [8]. Bands at 510 and 811 are solely seen in γ-phase samples and result from CH_2_ bending modes [7] and CH_2_ rocking modes of TTTG chain conformations [10]. The rise of intensities at 810 and 826 cm^−1^ are also attributed to the formation of the polar γ-phase [2]. The formation of the polar γ-phase is expected in LVS samples considering the process parameters (Table 2). Kobayashi et al. prepared pure γ-phase samples from dimethylacetamide solution [8], differing only by one CF_3_ group to DMF.

**Figure 4:**
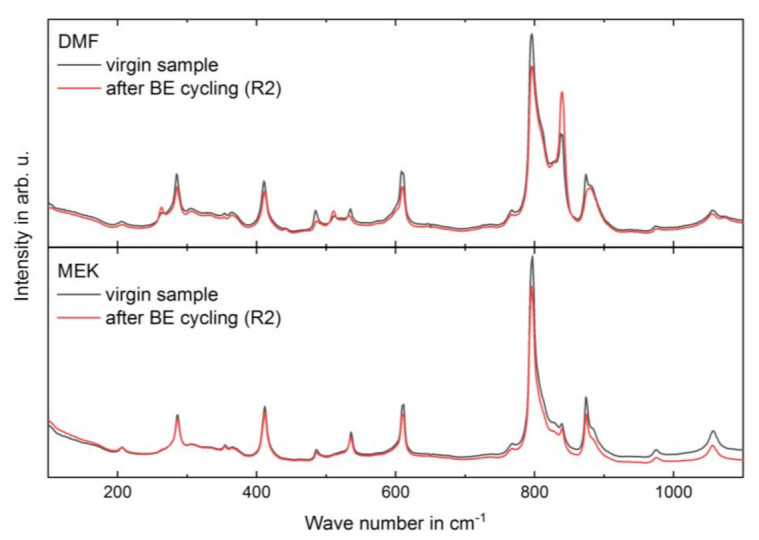
Raman spectra of virgin sample (black) and sample after two runs of BE cycling (red) of films processed from solvents DMF (**top**) and MEK (**bottom**).

Raman spectra of copolymer films measured within a few hours after two runs of high field bipolar electrical (BE) cycling (for more detail on BE cycling, see section Electrical characterization and section High-electric-field *D-E* characteristics) are shown in Figure 4 and compared to the spectra of virgin films. BE cycling leads to an increase in Raman intensities of polar γ-phase bands for samples from DMF solution and no significant changes for samples from MEK solution. The change in phase composition is considered via the band intensity at 840 cm^−1^ normalized to 795 cm^−1^, characteristic for polar and nonpolar phase, respectively (Table 5). Regarding absolute intensity values of the Raman spectra, the intensity of characteristic polar γ-phase bands increases in BE treated samples from DMF solution. Simultaneously, intensities of characteristic nonpolar α-phase bands decrease.

### 3.2. High-Electric-Field D-E Characteristics

#### 3.2.1. Breakdown Strength

For estimating the characteristic breakdown strength (BDS), eight contacts are examined for each sample. The BDS is above 400 MV/m for all films using a two-parameter Weibull analysis [29,41]. Films from acetone and MEK solutions show the highest and lowest BDS of 525 MV/m and 405 MV/m, respectively. The BDS of the LVS samples is at 435 MV/m and 460 MV/m for samples from NMP and DMF solutions, respectively. The accuracy of the measurement is around 10% (caused by thickness uncertainty). The different structural characteristics of the samples appear to have no influence on the breakdown field strength of the films.

#### 3.2.2. Bipolar *D-E* Characteristics

In the following, MEK and DMF samples are analyzed as representatives for HVS and LVS, respectively. The FE behavior of the samples was investigated by bipolar electric displacement field-electric field (*D-E*) loops to determine the remanent polarization and the coercive field *E*_c_; current density-electric field (*j*-*E*) loops (*j* = $\dot{D}$) were also used to verify FE polarization switching. Cycling electric fields up to a maximum amplitude of 283 MV/m corresponds to 70% of the lowest characteristic BDS of the samples to avoid dielectric breakdown were applied. The BE cycling run was applied two times immediately, one after another. R1 refers to BE cycling run 1, i.e., the measurement of the virgin sample, while R2 is the repeated measurement. In order to observe a state as virgin as possible, the 2nd cycle of R1 was considered and evaluated for further interpretation. In R2, the 9th cycle was considered. The *D-E* curve gap at zero field *D*_split_ was two times the value of remanent polarization originating in FE polarization reversal and retention with twice the FE remanent polarization. Additionally, the imbalance of further polarization and depolarization processes, as well as conduction processes, contributes to *D*_split_ [42,43,44,45,46]. The conductivity influence can be recognized in *j-E* curves by nonconstant currents even after the FE switching peak and during discharge (as it should for a triangular field sweep) [44,45]. FE behavior can be clearly identified by an FE switching peak in the *j-E* curves and only conditionally by *D*_split_ due to the mentioned contributions to *D*_split_.

**Figure 5:**
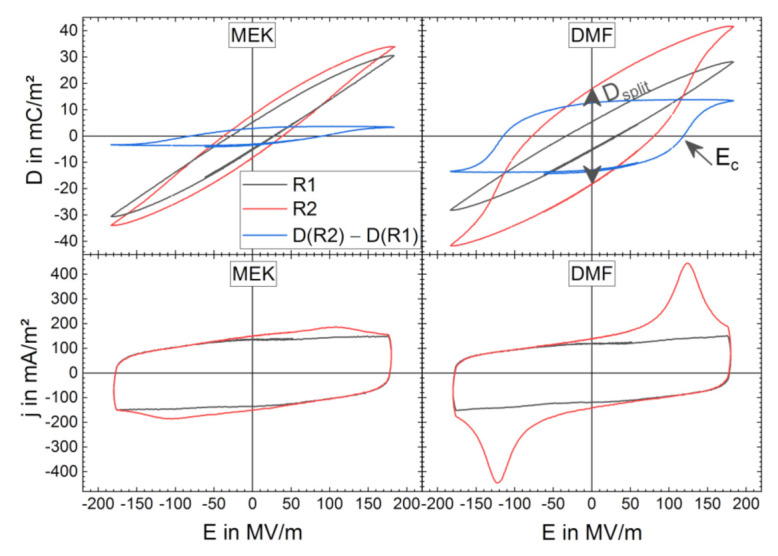
Bipolar electric displacement field *D* vs. electric field *E* (**top**) and corresponding current density j vs. electric field E (**bottom**) loops of BE cycling run 1 (R1) (black lines) and BE cycling run 2 (R2) (red lines) for films from MEK (**left**) and DMF (**right**) solutions with a cycling field amplitude maximum of *E*_ampl_ = 183 MV/m. The difference of *D-E* loops of R2 and R1 (blue lines) are shown, and the *D-E* curve gap at zero field *D*_split_ and position of coercive field *E*_c_ are indicated for the DMF sample.

The *D-E* loops of virgin films from the MEK and DMF solutions (Figure 5) have an almost identical shape and show non-FE behavior in R1 with the cycling field amplitude maximum *E*_ampl_ up to 183 MV/m since there is no peak in the *j-E* curves indicating FE polarization reversal (*D-E* and *j-E* curves of all field amplitudes are provided in Figures S3 and S4). Cycling at fields above 183 MV/m (up to 283 MV/m) leads to a *j-E* peak build-up and a distinct FE hysteresis forming for both samples in R1 (see Figures S3 and S4). Already with lower *E*_ampl_, a FE behavior is observed in R2. The development of FE behavior is more pronounced in the DMF sample with a higher displacement field maximum as well as a higher FE switching current peak compared with the MEK sample. The *D-E* loop of R1 is subtracted from the *D-E* loop of R2 to determine *E*_c_. The difference loop (see blue lines in Figure 5) exclusively presents the ferroelectric polarization hysteresis without further contributions to *D*, similar to the conduction current or dielectric polarization that deform the hysteresis. *E*_c_ is about 93 MV/m and 118 MV/m for the samples from MEK and DMF, respectively. The values for *E*_c_ are mean values of the intercepts in the negative and positive field range.

**Figure 6:**
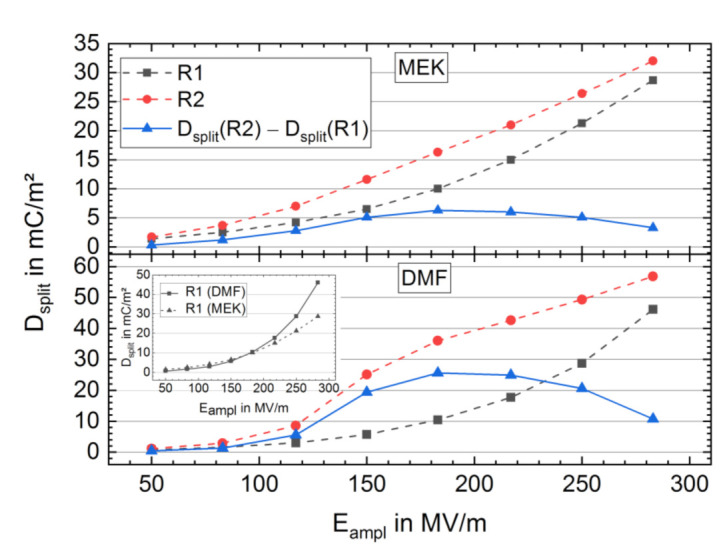
*D-E* curve gap at zero field *D*_split_ vs. cycling field amplitude maximum *E*_ampl_ of BE cycling run 1 (R1) (black) and BE cycling run 2 (R2) (red) for samples from MEK (**top**) and DMF (**bottom**) solution. Additionally,$\;\Delta D_{split}^{21}$ = *D*_split_ (R2) − *D*_split_ (R1) is shown (blue). The inset compares the two samples in BE cycling run 1 (R1).

*D*_split_ of R1 and R2 as a function of *E*_ampl_ is shown in Figure 6. The courses of *D*_split_ in R1 are almost congruent up to *E*_ampl_ = 183 MV/m for DMF and MEK samples (see inset in Figure 6), and *D*_split_ rises non-linearly. Above *E*_ampl_ = 183 MV/m, the DMF sample shows a stronger increase in *D*_split_, and a peak in the *j-E* curves starts forming (see Figure S4), indicating a structural change in the samples and the formation of FE switching activity. In R2, a clear FE switching behavior with a typical stepwise increase in *D*_split_ around *E*_c_ appears, more pronounced for the sample from DMF. The highest applied field amplitude of 283 MV/m *D*_split_ of R1 and R2 closely came together, indicating nearly a saturation of structural changes within R1 (see also Figure S7 for *D*_split_ vs. *E*_ampl_ depending on cycling number). The difference $\Delta D_{split}^{21}$ = *D*_split_ (R2) − *D*_split_ (R1) can be used to extract the contribution of FE switching to *D*_split_ (R2), as far as FE switching in R1 is neglected. In $\Delta D_{split}^{21}$ all contributions to *D*_split_ (R2) besides FE polarization reversal are eliminated, assuming they are the same in R1 and R2. $\Delta D_{split}^{21}$ increases till *E*_ampl_ = 183 MV/m. We regard the peak value as twice the maximum FE remanent polarization of the poled samples in R2 formed due to an increase in FE switching active dipoles from the polar crystalline phase during R1. This is a good approximation, as no significant FE switching occurs in R1 until *E*_ampl_ = 183 MV/m (no peak in *j-E* curve, see Figure 5), FE reversal in R2 at this field strength is almost complete (see FE switching peak width in *j-E* curve of Figure 5) with the assumption that no further growth of FE remanent polarization in R2 occurs at higher field strengths than 183 MV/m. The peak value of $\Delta D_{split}^{21}$ for *E*_ampl_ = 183 MV/m is approximately 25.6 mC/m^2^ (maximum FE remanent polarization of 12.8 mC/m^2^) for the sample from the DMF solution, which is four times higher than the sample from MEK with a $\Delta D_{split}^{21}\;$peak value of 6.2 mC/m^2^ (maximum FE remanent polarization of 3.1 mC/m^2^). For *E*_ampl_ higher than 183 MV/m, $\Delta D_{split}^{21}$ decreases, which can be associated with enhanced structural changes in the virgin samples leading to a gradual increase in the FE remanent polarization contribution to *D*_split_ (R1).

The discharge (*w*_d_) and charge (*w*_ch_) energy densities, as well as the discharge efficiency, are directly obtained by integration from the *D-E* loops (*w*_d/ch_ = ∫*E*_d/ch_ d*D*) of R1 and R2 for *E*_ampl_ = 183 MV/m. *E*_d_ (*D*) is the discharge, and *E*_ch_ (*D*) the charge branch of the electric field. The discharge efficiency (*η*) is defined as *η* = *w*_d_/*w*_ch_. *w*_d_ for R1 and R2 is about 1.95 MJ/m^3^ and 1.77 MJ/m^3^ for films from MEK and DMF, respectively. The uncertainty in *w*_d_ of the two films is 10% ascribed to the film thickness uncertainty. There is no significant difference in the discharge energy densities of the samples. There is also no significant influence in the increased FE remanent polarization or the changed sample character from non-FE to FE behavior by BE cycling on the discharge energy density. Nevertheless, *w*_ch_ and *η* increase and decrease, respectively, from R1 to R2 due to the increase in FE remanent polarization, and in a stronger manner for the sample from DMF compared with the sample from MEK because FE remanent polarization is increased for the former. In R1, *w*_ch_ is approximately 3.31 MJ/m^3^ and 3.16 MJ/m^3^; in R2, 3.9 MJ/m^3^ and 6.14 MJ/m^3^ for films from MEK and DMF, respectively. *η* of the DMF sample decreases from 56% to 29% whereas only a small decrease of 59% to 50% is observed for the sample from MEK.

### 3.3. Long-Term Stability of the BE Treated Samples

After storing BE treated (R2) samples with a removed electric field at room temperature in the dark for 4 months, the structural characterization and investigation of the FE behavior were repeated to analyze the temporal stability of BE cycling-induced changes.

**Figure 7:**
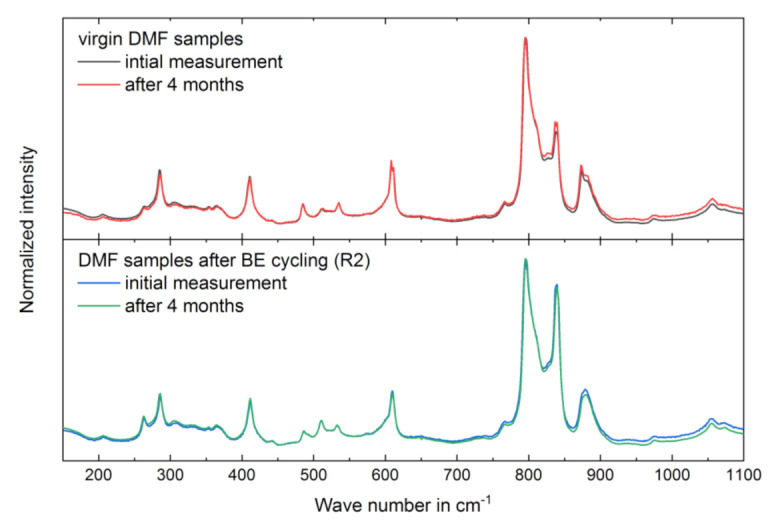
Raman spectra of samples from DMF solution. Virgin samples (without BE cycling) (**top**): initial (black) and repeated measurement (red). BE cycled samples (**bottom**): initial measurement (blue) after BE cycling (R2) and repeated measurement (green). Repeated measurements were conducted 4 months after the initial measurements (see Materials and Methods section).

Raman measurement was performed again on the virgin and BE treated (R2) samples. The Raman spectra are in good agreement with the measurements before sample storage (Figure 7). Temporal stability investigations are only shown for DMF samples. The MEK samples did not show significant differences in Raman spectra before or after the BE cycling and storage period. The intensity ratio at 840 cm^−1^ and 795 cm^−1^ after storage was 0.54 for the virgin DMF sample and 0.86 for the BE treated (R2) DMF sample. These results are in good agreement with the values before storage and indicates the stability of the field-induced structural change (compare with Table 5).

**Figure 8:**
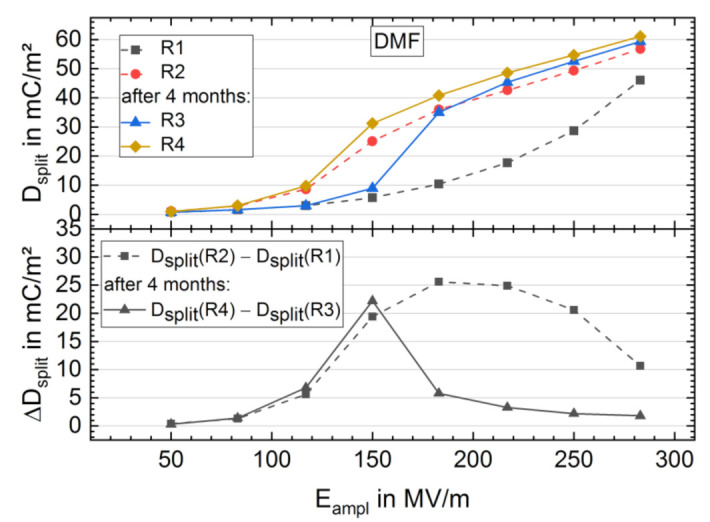
*D-E* curve gap at zero field *D*_split_ vs. cycling field amplitude maximum *E*_ampl_ of BE cycling run 1 to 4 (R1 to R4) for a sample from DMF solution (**top**). Between BE cycling Run 2 and 3, the sample was stored for 4 months (see Materials and Methods section). Additionally, the difference in *D*_split_ vs. *E*_ampl_ of R2 and R1 (dashed line) and R4 and R3 (solid line) is shown (**bottom**).

After sample storing, the investigation of FE behavior was performed by two further BE cycling runs (R3 and R4) in an equal manner as for R1 and R2 on BE treated (R2) contacts. In the following analysis, only the DMF sample (Figure 8) is regarded with a stronger effect (see Figure S8). In R3, the FE behavior appears only for cyclisation with an *E*_ampl_ of 150 MV/m or higher, similar to the behavior in R1, which is higher than *E*_c_ with 118 MV/m in R2 (see Figure 8, Figures S5 and S6). *D*_split_ of R3 abruptly rises for *E*_ampl_ higher than 150 MV/m, reaches the value of *D*_split_ of R2 at approximately 183 MV/m and runs parallel to *D*_split_ of R2 for higher fields. *D*_split_ of R3 even slightly exceeds *D*_split_ of R2 with higher *E*_ampl_, indicating an additional increase in FE switching active dipoles from the polar crystalline phase. The course of *D*_split_ of R4 directly after R3 has almost the same shape as that of *D*_split_ in R2, merely showing higher values of approximately 5 mC/m^2^ for *E*_ampl_ exceeding *E*_c_, as shown in Figure 8. Figure 8 shows that $\Delta D_{split}^{21}$ and $\Delta D_{split}^{43}$ = *D*_split_ (R4) − *D*_split_ (R3) versus *E*_ampl_. $\Delta D_{split}^{43}$ has a narrower peak with a maximum at a lower *E*_ampl_ than the peak in $\Delta D_{split}^{21}$; this is due to the faster increase in FE remanent polarization at a lower *E*_ampl_ for the sample in R3 than in R1. Left curve flanks are almost congruent until the peak maximum of $\Delta D_{split}^{43}$ at an *E*_ampl_ of 150 MV/m, implying that the same process in R1 and R3 until this field strength.

## 4. Discussion

The polymer film surface structure and the crystalline phase composition of P(VdF-HFP) films are influenced by the use of different solvents. With HVS (MEK, acetone), smooth film surfaces (12–14 nm RMS roughness) with homogeneously distributed crystalline lamellae can be obtained, whereas LVS (DMF, NMP) produces a rough surface (22–33 nm RMS roughness) with large spherulite structures (see Figure 1 and Figure S2); this is due to the longer crystallization time and the increased mobility of polymer chains at a higher crystallization temperature for films from LVS. DSC measurements reveal that the crystallinity of the HVS samples is approximately 7% points higher (with an absolute crystallinity of 43%) than the LVS samples attributed to the mass crystallization effect. Samples from HVS contain predominantly P(VdF) α-phase crystals, whereas samples from LVS have a large proportion of P(VdF) γ-phase (Figure 3, left). The phase identification is based on XRD and Raman measurements. According to the former method, the existence of a P(VdF) β-phase has been excluded. The formation of γ-phase in the copolymer LVS films with a higher drying temperature and a longer drying time (see Table 2) is consistent with the well- known observation of α- to γ-phase transition for annealed P(VdF) films, as the γ-phase is the thermodynamically favored form at elevated temperatures [46]. Despite the differences in the films’ phase composition depending on the solvent used, especially the high amount of γ-phase in LVS films, all samples show non-FE behavior until *E*_ampl_ = 183 MV/m.

The BE cycling at high electric fields of samples from MEK and DMF solvents, as representatives for HVS and LVS, respectively, leads to a change from non-FE to FE behavior, which is more pronounced in the DMF sample (see Figure 5 and Figure 6). Additionally, a change in the Raman spectrum of the sample from DMF occurs, showing an increased intensity of bands in the polar phase compared with nonpolar phase bands. In the following list, different possible reasons for the field-induced changes are discussed:

1. A shielding effect that prevents dipoles of existing polar phase from polarization reversal during the BE cycling;
2. The formation of further polar phase from amorphous phase or via a crystalline phase transition;
3. The reorientation of dipole moments and cooperative rotation of the chain axes in the existing polar phase.

The non-FE behavior mentioned for the virgin samples, despite the high amount of the γ-phase in the DMF sample, can be attributed to a shielding effect in the virgin samples that blocks the FE dipole reversal of the polar domains [47]. The shielding effect can be caused by the amorphous phase at the interface of the crystalline polar phase. This effect has been described by Peter and Kliem as an imprint effect and was investigated in films of P(VdF-TrFE) [47]. Dipoles of the amorphous phase are expected to align in the direction of the stray field of polar domains antiparallel to the dipoles of the polar phase domain. The aligned dipoles of the amorphous phase itself jointly evoke a stray field, the imprint field, that stabilizes the ferroelectric polarization state of the surrounding domains and increases necessary field strength for polarization reversal [47]. By BE cycling at fields higher than 180 MV/m, the shielding effect can be overcome and reduced, and both samples show FE behavior (see Figure S4). In an immediately repeated BE cycling run, FE behavior was already seen at lower fields with *E*_c_ at 93 MV/m and 118 MV/m for the sample from MEK and DMF, respectively. The rise of FE remanent polarization from a virgin to BE treated state is four times higher in the DMF (12.8 mC/m^2^) than in the MEK (3.1 mC/m^2^) sample (see Figure 6). The smaller FE remanent polarization is likely due to the smaller amount of polar γ-phase in the virgin MEK sample (see Figure 3).

Due to the BE cycling with *E*_ampl_ up to 283 MV/m, the Raman intensities of the γ- in relation to the α-phase bands increase strongly for the sample from DMF, whereas the spectra of the MEK sample show no significant change (see Figure 4 and Table 5). This observation for the sample from DMF indicates that in addition to the shielding effect, the formation of a new polar phase or the reorientation of the existing polar phase occurs. No further investigations were performed to differentiate between these two processes. In the Raman spectrum of the DMF sample, we also see a slight reduction of α-phase bands after two runs of BE cycling, suggesting a phase transition from α- to γ-phase (see Figure 4). The formation of a new polar phase by solid-state phase transition from the α- to γ-phase and the underlying mechanism was studied by Lovinger et al. [48] and Takahashi and Tadokoro [49]. The transition is mainly reported for thermally treated samples [20,21,50], but there are indications for field-induced transitions from α- to polar β- or γ-phase [38,51,52]. For the field-induced formation of the γ-phase by BE cycling, crystallization nuclei of the γ-phase are needed in the virgin sample [9]. As the amount of the γ-phase is lower and crystalline superstructures like spherulites do not exist in the MEK sample (see Figure 1), only little interaction of γ-phase domains with neighboring domains occurs; this possibly hinders a field-induced formation of the γ-phase in the MEK sample.

Crystalline domains within the virgin samples are supposed to be randomly oriented, and in each polar crystallite, the dipole moments are randomly oriented in a plane normal to the chain axis. When an electric field is applied, the CH_2_-CF_2_ dipoles in each polar crystallite rotate about their chain axes to align toward the field direction as demonstrated by various authors [46,53,54]. A cooperative rotation of the chain axes accompanied by the dipole reorientation is also reported [55], where chain axes tend to reorient in a plane perpendicular to the applied field that would further increase the scalar product of the dipole moments with the electric field, leading to an increase in maximum and remanent polarization [53,56].

A field-induced formation of crystalline polar phase from the amorphous phase or polymer chains in the interfacial layer between the crystalline and amorphous regions is discussed for poly(vinylidene fluoride-trifluoroethylene) (P(VDF-TrFE)) [57,58,59]. A gradual increase in the ordering degree of the interfacial layer has been suggested as a contributing factor to the polarization (maximum and remanent) enhancement during electric cycling and the gradual growth of crystallite size. Mabuchi and coworkers assumed that the increase in remnant polarization and the P(VdF) β-phase diffraction peak during BE cycling of P(VDF-TrFE) films is due to an induced crystallization by a molecular alignment of the amorphous phase [60]. The following evaluation of our results, however, shows no hint of a field-induced increase in materials crystallinity (as a result of the formation of crystalline phase from amorphous phase).

The discharge energy density of the samples from DMF and MEK show no significant change after BE cycling related to a transition from non-FE to FE behavior; this implies that the process leading to the transition from non-FE to FE behavior and the altered Raman spectrum does not affect the materials dielectric polarization mechanisms, hence their relative permittivity *ε*_r_. In general, in materials with permanent dipoles, *ε*_r_ of the amorphous phase is higher than *ε*_r_ of the crystalline phase because the crystalline phase delivers no orientation polarization contribution. From the unchanged energy density, the amount of amorphous and crystalline phases is assumed to stay constant.

In a simple estimation, we determined the necessary minimal fraction of VDF monomers in the samples arranged in the γ-phase to generate the measured FE remanent polarization of the BE treated (R2) samples (see estimations in Supplementary Materials). Bearing in mind the assumption that all dipole moments of the γ-phase fully contribute to the FE remanent polarization (dipoles are oriented in parallel to the excitation field) at a minimum of 3% and 12% of the VDF, monomers must be arranged in the γ-phase in samples from MEK and DMF, respectively. Comparing these values to the crystallinity of the virgin samples (where the crystallinity presents the VDF monomer fraction in a crystalline phase; crystallinity is determined from DSC measurement), it follows that 7% and 33% of the crystalline phase must be arranged in γ-phase conformation for the samples from MEK and DMF, respectively. Therefore, the FE remanent polarization built up due to high-electric-field BE cycling can be explained without the necessity of crystalline phase growth by the aforementioned processes.

Repeated structural characterization and investigation of FE behavior after a sample storing time of 4 months supports the assumption of two mechanisms taking place during high-electric-field BE cycling in the DMF sample. On the one hand, the ratio of γ-phase to α-phase Raman band intensities remains unchanged (Figure 7), indicating a temporal stable crystal phase composition or stable polar phase domain alignment induced by high-electric-field BE cycling. On the other hand, the electric field for FE dipole switching increased to approximately 150 MV/m after sample storing, and samples show no FE behavior at lower fields, similar to the behavior of the virgin samples. The shielding effect, hindering FE dipole reversal, appears to rebuild with time, as discussed by Peter and Kliem [47]. They found a linear increase in ferroelectric polarization reversal field strength as a function of the logarithm of time after the previous polarization reversal. For fields higher than approximately 180 MV/m, the maximum FE remanent polarization is already reached, whereas the FE remanent polarization for the virgin samples continues to rise for fields up to approximately 280 MV/m (Figure 8). Therefore, the formation of further polar phase or the reorientation of the existing polar phase occurring at electric fields of more than approximately 180 MV/m and overcoming the shielding effect occurring at electric fields up to approximately 180 MV/m in the investigated P(VDF-HFP) samples is assumed.

## 5. Conclusions

The choice of the solvent for producing P(VdF-HFP) films via the solution casting method is crucial for the crystalline structure and morphology. The main factors are depending on the evaporation time of the solvents and film drying temperature. HVS leads to predominantly nonpolar α-phase crystals with a smooth film surface, while LVS leads to a high fraction of polar γ-phase crystals and a rough film surface. Independent from phase composition, the P(VdF-HFP) films show non-FE behavior. A shielding effect hinders dipole moments of polar crystalline phase domains from polarization reversal. This shielding can be overcome and reduced by high-field cyclic BE cycling leading to an FE-behavior of the films and a gradual reduction in necessary field strength for dipole switching. With dwell time, the shielding rebuilds. Additionally, the BE cycling at even higher fields up to approximately 300 MV/m appears to induce a growing γ-phase fraction through crystalline phase transition or alignment of existing γ-phase domains.

## Figures and Tables

**Table 1:** Properties of the different solvents used for fabricating polymer films. Values for boiling point, vapor pressure and density are taken from the manufacturer’s data.

Solvent	Boiling Point in °C	Vapor Pressure at 20 °C in hPa	Density at 20 °C in g/cm^3^
Acetone (HVS)	56	244	0.79
MEK (HVS)	80	105	0.81
DMF (LVS)	153	3.77	0.95
NMP (LVS)	202	0.32	1.03

**Table 2:** Adjusted parameters of polymer solution drying process in the doctor blade coating machine.

Solvent	Temperature of Process Ta- ble in °C	Intensity of Infra-Red Lamp in % of 1 kW	Temperature of Convection Dryer in °C	Time When Samples Ap- pear Optically Dry in s	Total Dwell Time under IR-Lamp in min
Acetone (HVS)	25	25	25	10	1
MEK (HVS)	40	25	40	20	1
DMF (LVS)	80	100	60	60	5
NMP (LVS)	80	100	60	360	10

**Table 3:** Properties of different solvents used for the fabrication of the polymer films. Values for boiling point, vapor pressure, and density are taken from the manufacturer’s data.

Solvent	Surface Rough- ness (RMS) in nm	Crystalline P(VdF) Phase	BDS in MV/m	Energy Density in MJ/m^3^
Acetone (HVS)	14	α	525 (±10%)	–
MEK (HVS)	12	α	405 (±10%)	1.95 (±10%)
DMF (LVS)	22	α + γ	460 (±10%)	1.78 (±10%)
NMP (LVS)	34	α + γ	435 (±10%)	–

**Table 4:** Ratios for Raman band intensities at 840 cm^−1^ and 795 cm^−1^, characteristic for polar and nonpolar crystalline structure in P(VdF), respectively.

Solvent	I (840 cm^−1^)/I (795 cm^−1^)
Acetone (HVS)	0.21 ± 0.01
MEK (HVS)	0.24 ± 0.02
DMF (LVS)	0.54 ± 0.06
NMP (LVS)	0.72 ± 0.12

**Table 5:** Ratios for Raman band intensities at 840 cm^−1^ and 795 cm^−1^, characteristic for polar and nonpolar crystalline structure in P(VdF), respectively.

Solvent	Electrical Treatment	I (840 cm^−1^)/I (795 cm^−1^)
MEK (HVS)	virgin sample	0.18 ± 0.02
	after BE cycling (R2)	0.21 ± 0.02
DMF (LVS)	virgin sample	0.53 ± 0.05
	after BE cycling (R2)	0.87 ± 0.13

## Data Availability

The data presented in this study are available on request from the corresponding author.

## Supplementary Materials

The following are available online at https://www.mdpi.com/article/10.3390/ma14143884/s1 Table S1: Solubility properties of used solvents, Figures S1 and S2: Atomic force microscopy (AFM) images of the P(VdF-HFP)*;* Figures S3–S6: *D-E* and *j*-*E* loops of P(VdF-HFP) films produced from MEK and DMF solutions for R1 to R4; Figure S7: *D*_split_ as a function of cycling electric field amplitude maximum and cycle number for R1 to R4 for the P(VdF-HFP) film produced from DMF solution; Figure S8: *D*_split_ and Δ*D*_split_ as a function of cycling electric field amplitude maximum for R1 to R4 for the P(VdF-HFP) film produced from MEK solution. Calculation details: determination of the minimal necessary fraction of VDF monomers in the samples arranged in the γ-phase to generate the measured FE remanent polarization of the BE treated (R2) samples.
