# Supplementary material for: Influence of Different Solvents and High-Electric-Field Cycling on Morphology and Ferroelectric Behavior of Poly(Vinylidene Fluoride-Hexafluoropropylene) Films
Source: Materials (Basel). 2021 Jul 12;14(14):3884. doi: 10.3390/ma14143884 (PMC8303348; doi:10.3390/ma14143884)
Supplement: Supplementary file 1 [file materials-14-03884-s001.zip › materials-1257490-supplementary.pdf]

materials

MDPI

Supplementary Materials

Influence of Different Solvents and High-Electric-Field Cycling on Morphology and Ferroelectric Behavior of Poly(Vinylidene Fluoride-Hexafluoropropylene) Films

Till Mälzer [1,2,*,†], Lena Mathies [1,†], Tino Band [3], Robert Gorgas [2] and Hartmut S. Leipner [1]

1 Interdisciplinary Center of Materials Science, Martin Luther University Halle-Wittenberg, Heinrich-Damerow-Straße 3, 06099 Halle, Germany; lena.kuske@cmat.uni-halle.de (L.M.); hartmut.leipner@cmat.uni-halle.de (H.S.L.)
2 enspring GmbH, Weinbergweg 23, 06120 Halle, Germany; robert169@web.de
3 Institute of Physics, Martin Luther University Halle-Wittenberg, Von-Danckelmann-Platz 3, 06099 Halle, Germany; tino.band@physik.uni-halle.de
* Correspondence: till.maelzer@cmat.uni-halle.de
† These authors contributed equally.

Citation: Mälzer, T.; Mathies, L.; Band, T.; Gorgas, R.; Leipner, H.S. Influence of different solvents and high-electric-field cycling on morphology and ferroelectric behavior of poly(vinylidene fluoride-hexafluoropropylene) films. *Materials* **2021**, *14*, x. https://doi.org/10.3390/

Academic Editor: Andrea Sanson

Received: 27 May 2021
Accepted: 6 July 2021
Published:

Publisher's Note: MDPI stays neutral with regard to jurisdictional claims in published maps and institutional affiliations.

Copyright: © 2021 by the authors. Licensee MDPI, Basel, Switzerland. This article is an open access article distributed under the terms and conditions of the Creative Commons Attribution (CC BY) license (http://creativecommons.org/licenses/by/4.0/).

Solubility properties of used solvents

The different solvation properties towards the copolymer especially for MEK are attributed to the different solubility parameters of the solvents (listed in Table S1). Solvents only solve the polymer if their solubility vector is within the Hansen ellipsoidal space with the three dimensions dispersion δ_d, polar δ_p and hydrogen δ_h bonding force [1–2]. Since solubility vector of MEK lies outside the solubility ellipsoid (see Table S1) a higher solution temperature is needed to solute the polymer.

Table S1. Solubility parameters of PVDF [2], acetone, MEK, DMF and NMP [1].

**Title**	δ_d	δ_p	δ_h	**Solubility parameter vector within (w)/outside (o) the PVDF solubility ellipsoid**[a]
PVDF	17.2	12.5	9.2	–
Acetone	15.5	10.4	7	0.73 (w)
MEK	16	9	5.1	1.35 (o)
DMF	17.4	13.7	11.3	0.28 (w)
NMP	18	12.3	7.2	0.32 (w)

[a] solubility parameter vector within the Hansen ellipsoidal space if ellipsoid equation $(\delta_{d,S} - \delta_{d,P})^2/7.7 + (\delta_{p,S} - \delta_{p,P})^2/64.4 + (\delta_{h,S} - \delta_{h,P})^2/17.2 \leq 1$, outside if > 1 [2] (with subscript P for polymer and S for solvent).

Atomic force microscopy (AFM) images of the P(VdF-HFP) films

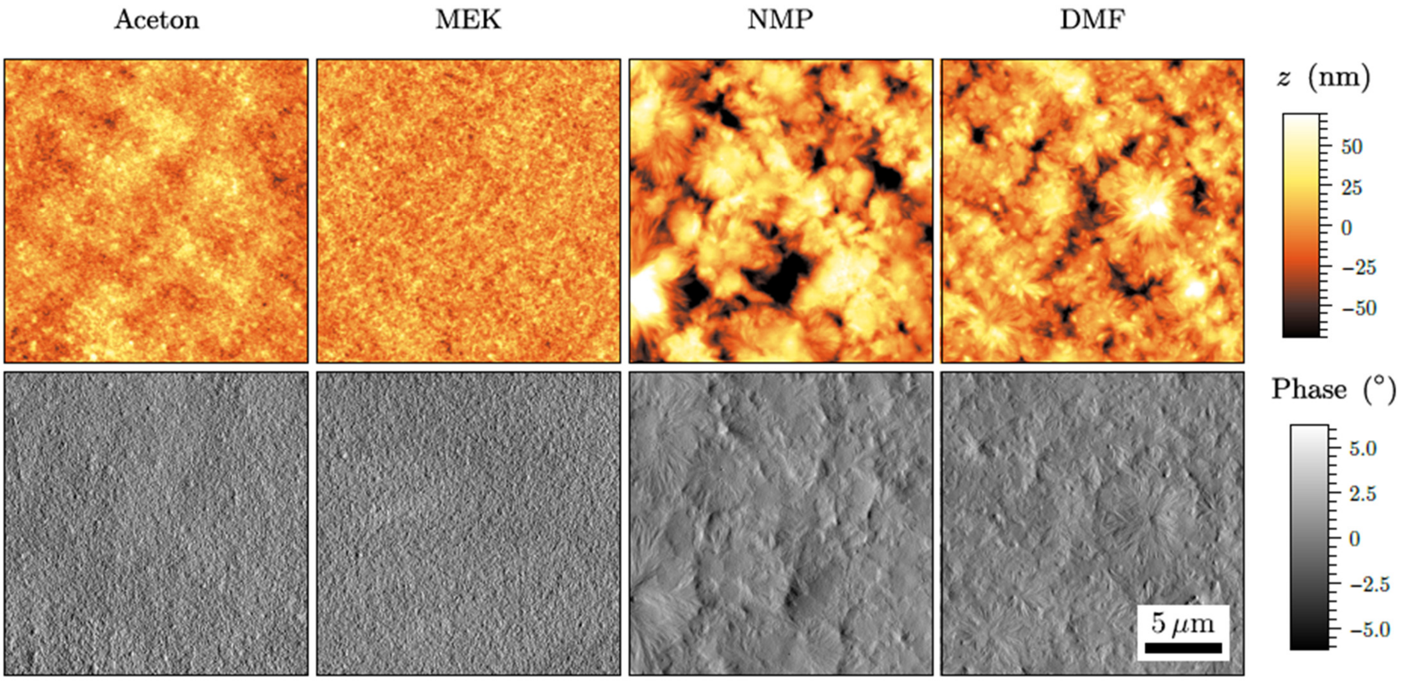

Figure S1. AFM topography height images (top) and phase images (bottom) of the films processed from different solvents.

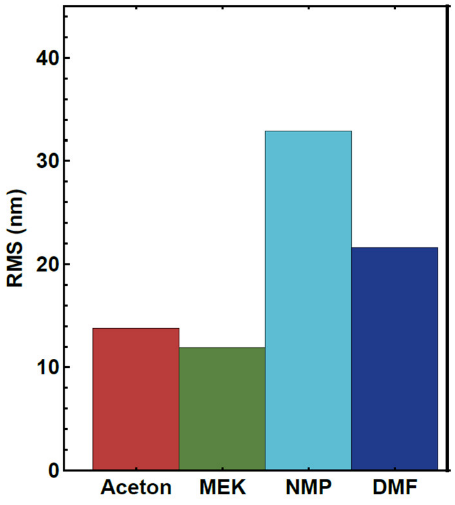

Figure S2. RMS roughness from AFM measurement of the films processed from different solvents.

D-E and j-E loops of P(VdF-HFP) films produced from MEK and DMF solutions for R1 to R4

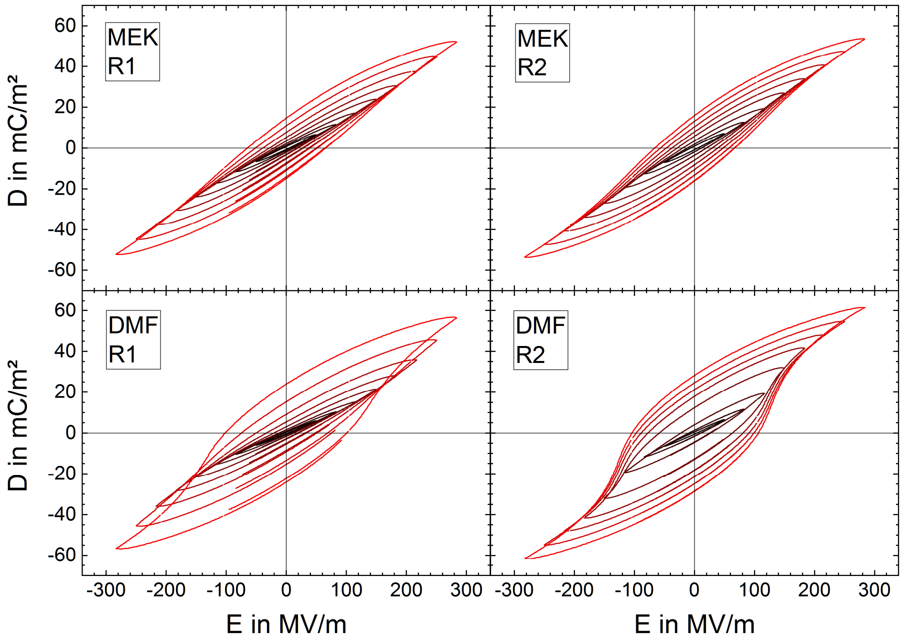

Figure S3. Bipolar electric displacement field *D* vs. electric field *E* loops of BE cycling run 1 (R1) (left) and BE cycling run 2 (R2) (right) for films from MEK (top) and DMF (bottom) solutions.

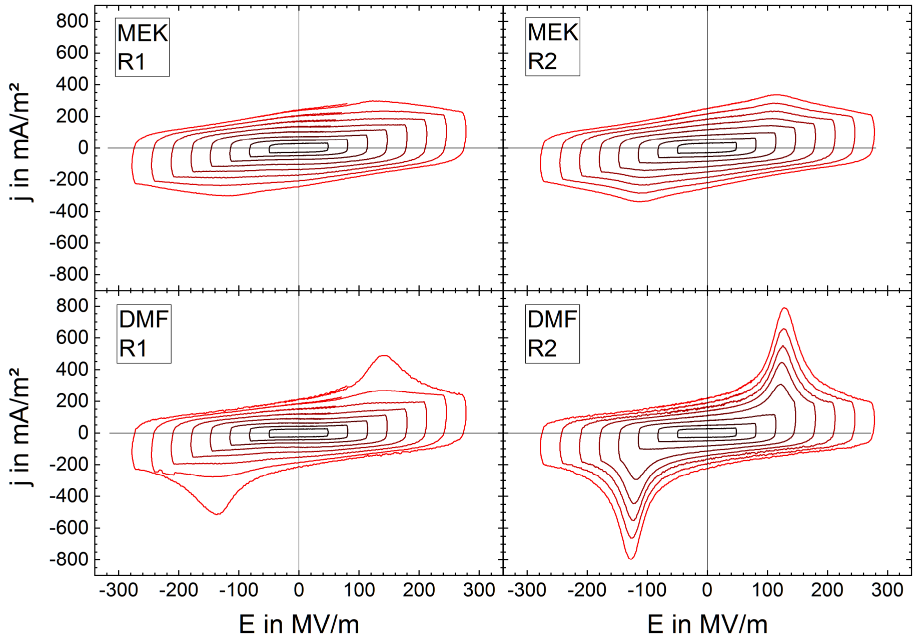

Figure S4. Current density *j* vs. electric field *E* loops (corresponding to *D*-*E* loops in Figure S3) of BE cycling run 1 (R1) (left) and BE cycling run 2 (R2) (right) for films from MEK (top) and DMF (bottom) solutions.

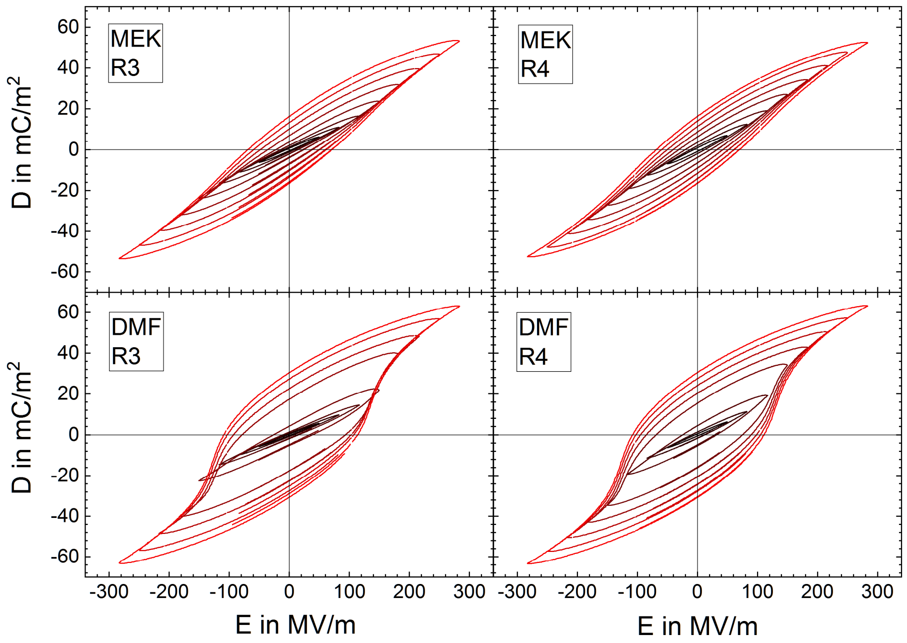

Figure S5. Bipolar electric displacement field *D* vs. electric field *E* loops of BE cycling run 3 (R3) (left) and BE cycling run 4 (R4) (right) for films from MEK (top) and DMF (bottom) solutions (BE cycling run 3 was conducted after storing the sample for 4 months subsequent to BE cycling run 2 (see Materials and Methods section)).

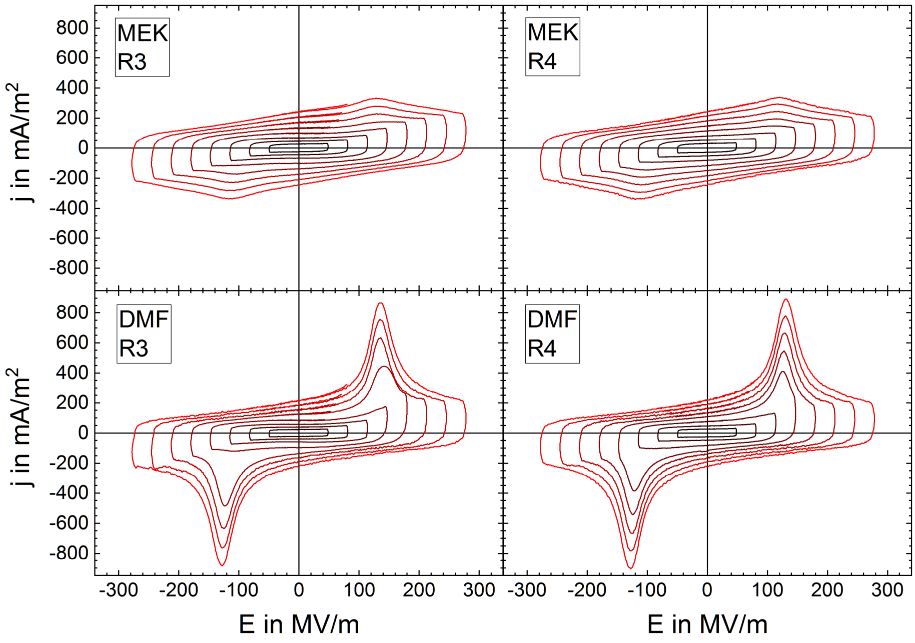

Figure S6. Current density *j* vs. electric field *E* loops (corresponding to *D*-*E* loops in Figure S5) of BE cycling run 3 (R3) (left) and BE cycling run 4 (R4) (right) for films from MEK (top) and DMF (bottom) solutions (BE cycling run 3 was conducted after storing the sample for 4 months subsequent to BE cycling run 2 (see Materials and Methods section)).

D_{split} as a function of cycling electric field amplitude maximum and cycle number for R1 to R4 for the P(VdF-HFP) film produced from DMF solution.

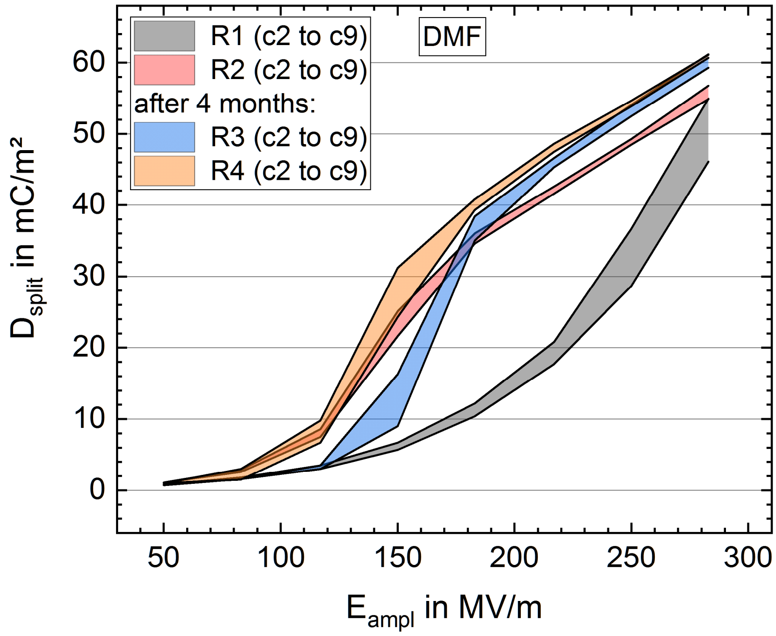

Figure S7. Range of *D*-*E* curve gap at zero field D_{split} vs. cycling field amplitude maximum E_{ampl} of BE cycling run 1 to 4 (R1 to R4) for a sample from DMF solution. D_{split} range limit values for each E_{ampl} correspond to D_{split} of cycle 2 (c2) and cycle 9 (c9) (with 10 cycles each E_{ampl} before increasing E_{ampl} (see Materials and Methods section)). Between BE cycling run 2 and 3 the sample was stored for 4 months (see Materials and Methods section).

D_{split} and ΔD_{split} as a function of cycling electric field amplitude maximum for R1 to R4 for the P(VdF-HFP) film produced from MEK solution.

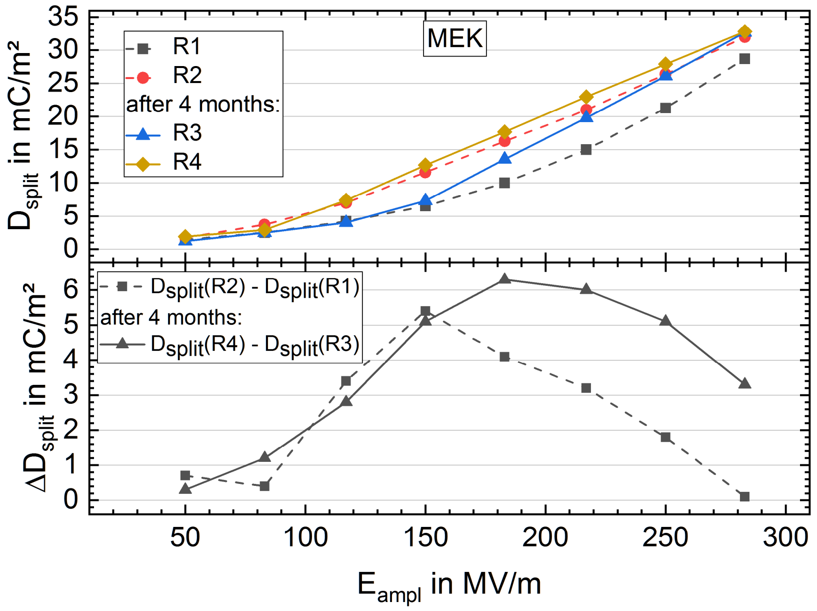

Figure S8. *D-E* curve gap at zero field D_{split} vs. cycling field amplitude maximum E_{ampl} of BE cycling run 1 to 4 (R1 to R4) for a sample from MEK solution (top). Between BE cycling run 2 and 3 the sample was stored for 4 months (see Materials and Methods section). Additionally, the difference in D_{split} vs. E_{ampl} of R2 and R1 (dashed line) and of R4 and R3 (solid line) is shown (bottom).

Calculation details

In this section we estimate the fraction of PVDF γ-phase responsible for the measured ferroelectric remanent polarization in BE treated samples.

Macroscopic polarization P of the copolymer at zero field can be expressed by the number N of permanent dipole moments μ of VDF monomers per sample volume V and the contribution of an additional moment the dipoles receive due to their polarizability α and the local electric field E_{loc} from the surrounding dipoles. It is assumed that the dipoles are aligned perfectly in parallel to the sample thickness and therefor contribute in maximum to P. It follows

$$P = \frac{N}{V}\mu + \frac{N}{V}\alpha\, E_{loc}. \quad (1)$$

The local field here is assumed to be composed only by the Lorentz field E_{Lor}, when sample electrodes are shorted together and local field from dipoles inside the Lorentz sphere is assumed to be zero [3–5]

$$E_{loc} = E_{Lor} = \frac{P}{3\varepsilon_0}.$$

With Clausius-Mossotti equation

$$\frac{\varepsilon_r - 1}{\varepsilon_r + 2}\frac{3\varepsilon_0 V}{N} = \alpha$$

with the permittivity of vacuum ε_0 and the relative permittivity ε_r (1) becomes to

$P = \frac{N}{V}\mu + \frac{\varepsilon_r - 1}{\varepsilon_r + 2}P$ and solving for P yields

$$P = \frac{N}{V}\frac{\varepsilon_r + 2}{3}\mu. \quad (2)$$

Since ferroelectric remanent polarization is induced by dipoles of the polar crystalline polymer phase, N is equal to the number of VDF monomers in this phase, called N_{pol}. Solving (2) for N_{pol} yields

$$N_{pol} = \frac{V}{\mu}\frac{3P}{\varepsilon_r + 2}. \quad (3)$$

The number of VDF monomers in the sample in general are composed by the number of monomers in the polar crystalline phase N_{pol}, the nonpolar crystalline phase N_{npol} and the amorphous phase N_{am}

$$N_{sample} = N_{pol} + N_{npol} + N_{am}. \quad (4)$$

With $\frac{N}{V} = \frac{N_A \rho_{av}}{M_{av}}$, with *Avogadro constant* N_{A}, the average molar mass M_{av} and the average sample density ρ_{av} (4) becomes to

$$N_{sample} = \frac{N_A \rho_{av} V}{M_{av}}. \quad (5)$$

For the ratio of monomers in the polar phase to the monomer number in the sample follows with (3) and (5)

$$\frac{N_{pol}}{N_{sample}} = \frac{M}{N_A \rho_{av} \mu}\frac{3P}{\varepsilon_r + 2}. \quad (6)$$

M_{av} = 66.3 g/mol as average molar mass of one VDF monomer + 0.027 HFP monomers (corresponding to 6 wt. % HFP in the copolymer), with molar mass of VDF of 64.0 g/mol and molar mass of HFP monomer of 150.0 g/mol. ρ_{av} = 1.77 g/cm^3 as manufacturer information, corresponding to a crystallinity of 0.4 with crystal phase density of 1.94 g/cm^3 [6], [7] and amorphous phase density of 1.68 g/cm^3 [8]). VDF monomer dipole moment μ in the VDF γ-phase of 4 x 10^{-30} Cm is assumed to be equal to monomer dipole moment in the δ-phase [4], [9] and be lower than the dipole moment in the β-phase with 7 x 10^{-30} Cm [4], [5], [10]. The low-field crystal relative permittivity $\varepsilon_r = 3$ [4]. The FE remanent polarization increase (P = ½ $\Delta D_{\mathrm{split}}$) (for cycling field amplitudes of 183 MV/m) in BE treated samples is 3.1 mC/m^2 and 12.8 mC/m^2 for samples from MEK and DMF solution, respectively.

With (6) it follows that 3 % and 12 % of the VDF monomers are arranged in the γ-phase conformation in the samples from MEK and DMF, respectively and are responsible for the FE remanent polarization. As crystallinity determined by DSC measurement or rather the VDF fraction in the crystalline phase is about 43 % and 36 % for samples from MEK and DMF solution, respectively, it follows that 7 % and 33 % of the crystalline phase needs to be arranged in γ-phase conformation for the samples from MEK and DMF, respectively.

It should be noted, that this estimation delivers a minimal value of γ-phase fraction in the samples especially due to the assumptions of perfectly aligned dipoles and defect-free crystals.

References

1. Hansen, C.M. *Hansen Solubility Parameters,* 2nd Edition, CRC Press, 2007, ISBN 9780429127526, p. 26.

2. Bottino, A.; Capannelli, G.; Munari, S.; Turturro, A. Solubility parameters of poly(vinylidene fluoride). *J. Polym. Sci. B Polym. Phys.* **1988**, *26*, 785–794, doi:10.1002/polb.1988.090260405.
3. Broadhurst, M.G.; Davis, G.T.; McKinney, J.E.; Collins, R.E. Piezoelectricity and pyroelectricity in polyvinylidene fluoride—A model. *Journal of Applied Physics* **1978**, *49*, 4992–4997, doi:10.1063/1.324445.
4. Kepler, R.G. Piezoelectricity, Pyroelectricity, and Ferroelectricity in Organic Materials. *Annu. Rev. Phys. Chem.* **1978**, *29*, 497–518, doi:10.1146/annurev.pc.29.100178.002433.
5. Furukawa, T. Ferroelectric properties of vinylidene fluoride copolymers. *Phase Transitions* **1989**, *18*, 143–211, doi:10.1080/01411598908206863.
6. *Developments in Crystalline Polymers-1*: *Poly(vinylidene fluoride)*. Andrew J. Lovinger; Bassett, D.C., Ed.; Springer: Dordrecht, 1982, ISBN 978-94-009-7345-9.
7. Hasegawa, R.; Takahashi, Y.; Chatani, Y.; Tadokoro, H. Crystal Structures of Three Crystalline Forms of Poly(vinylidene fluoride). *Polym J* **1972**, *3*, 600–610, doi:10.1295/polymj.3.600.
8. Nakagawa, K.; Ishida, Y. Estimation of amorphous specific volume of poly(vinylidene fluoride) as a function of temperature. *Kolloid-Z.u.Z.Polymere* **1973**, *251*, 103–107, doi:10.1007/BF01498933.
9. Lovinger, A.J. Annealing of poly(vinylidene fluoride) and formation of a fifth phase. *Macromolecules* **1982**, *15*, 40–44, doi:10.1021/ma00229a008.
10. Lovinger, A.J. Ferroelectric polymers. *Science* **1983**, *220*, 1115–1121, doi:10.1126/science.220.4602.1115.
